# Use of the 4K-3D ORBEYE Exoscope for Supermicrosurgical Lymphaticovenular Anastomosis: A Case Report

**DOI:** 10.7759/cureus.71198

**Published:** 2024-10-10

**Authors:** Anna Amelia Caretto, Stefano Gentileschi

**Affiliations:** 1 Plastic and Reconstructive Surgery, Fondazione Policlinico Universitario Agostino Gemelli IRCCS (Istituto di Ricovero e Cura a Carattere Scientifico), Rome, ITA

**Keywords:** 4k-3d exoscope, case report, digital imaging, lymphatic anastomosis, supermicrosurgery

## Abstract

The evolution in microsurgery using high-definition three-dimensional (3D) cameras has provided the opportunity to replace conventional operating microscopes (OM), improving ergonomics for microsurgeons. Several 3D exoscope systems have already demonstrated good surgical field visualization in a 3D space in performing microvascular anastomosis with favorable maneuverability and non-inferiority compared to OM. We present the application of the 4K-3D ORBEYE system (Olympus Inc., Tokyo, Japan) in performing lymphaticovenular anastomosis to treat lower limb lymphedema and the challenges and tips to manage small vessels measuring <0.8 mm. This system provided a good intraoperative outcome with greater ergonomics and a non-inferiority magnification compared to traditional microscopes. Our findings need further studies to confirm the feasibility; however, the 4K-3D ORBEYE represents a valid possibility to assist microsurgeons in lymphatic surgery.

## Introduction

Since the 1960s, surgical microscopes have always supported microsurgical activities by providing an enlarged view of the surgical field [1]. The limitations of standard models are mainly the 2D visualization and the need for a static, often non-neutral, posture of the surgeon during the procedure. This makes the microsurgeon particularly susceptible to developing cervical musculoskeletal problems, discomfort, and chronic pain [2,3]. New visualization systems that use digital imaging have been developed to replace traditional systems and overcome their limitations. Among these systems, the use of three-dimensional (3D) exoscopes has been popularized in various surgical areas for their ease of maneuverability and ergonomics, which frees the microsurgeon from the constraints of microscopes, loupes, or other magnification systems [4-7]. Previous reports have already demonstrated good visualization of the surgical field in a 3D space when performing microvascular anastomoses and non-inferiority compared to surgical microscopes [8-11]. Ichikawa et al. reported the use of the digital microscope Hawk Sight (Mitaka, Tokyo, Japan) in lymphaticovenular anastomosis (LVA) [12]. There is limited literature investigating the use of 3D exoscopes in LVA, and a paucity of discussion about the challenges of managing vessels smaller than 0.8 mm. In this article, we report the first experience in performing supermicrosurgical LVA using the 4K-3D ORBEYE system (Olympus Inc., Tokyo, Japan).

## Case presentation

A 65-year-old man presented with lymphedema in the left lower limb following pelvic lymphadenectomy for prostate cancer performed two years before. The patient reported two previous episodes of lymphangitis and a worsening of swelling over the past year. Lymphoscintigraphy demonstrated reduced visualization of the main lymphatic pathway, transport index of 14, and dermal backflow at the distal third of the left leg and in the ipsilateral inguinal region, suggesting lymphatic obstruction. Following the lymphoscintigraphic study, we decided to perform LVA on the patient.

**Figure 1:**
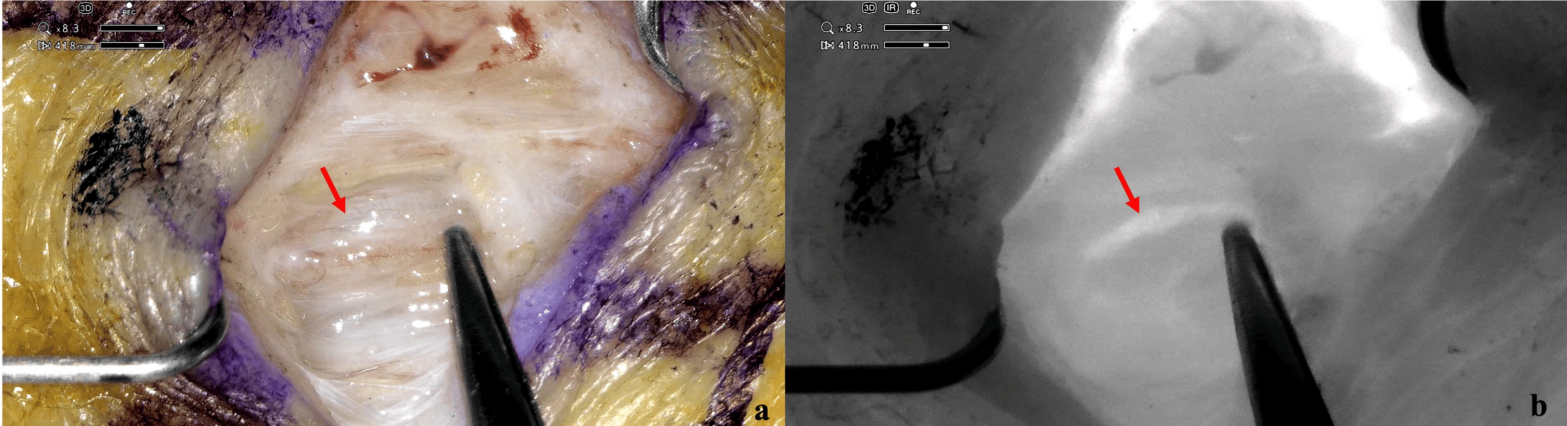
Intraoperative picture of lymphatic vessels with ORBEYE system. (a) An intraoperative picture during surgery shows an ectasic lymphatic vessel (red arrow) in the hypodermic layer at the anterolateral surface of the left leg. (b) After ICG (indocyanine green) injection, the infrared mode of ORBEYE system confirmed the good function of the lymphatic vessel, indicated by a red arrow.

The day before the surgery, the patient underwent indocyanine green lymphography (ICG-L) and color Doppler ultrasound (CDU) to respectively map the position of functioning lymphatic vessels and adjacent venules without blood reflux. The lymphography was performed by injecting intradermally 0.2 ml of indocyanine green (ICG) (Verde Indocianina Pulsion, Pulsion Medical Systems AG, Munich, Germany) at the first interdigital space of the foot and 0.2 ml immediately posterior to the lateral malleolus to identify the course of lymphatic vessels and the pattern of dermal backflow using a handheld infrared camera (Fluobeam, Fluoptics, France). The initial phase of the ICG-L demonstrated a splash pattern with compensatory ectasic lymphatic vessels, while the late phase revealed a stardust pattern. We planned three incision sites for anastomosis: on the anterolateral surface of the leg, at the distal third of the thigh, and in the groin, following a technique described in our previous report [13]. At each site, we found ectasic lymphatic vessels near venules without blood reflux (Figure 1).

**Figure 2:**
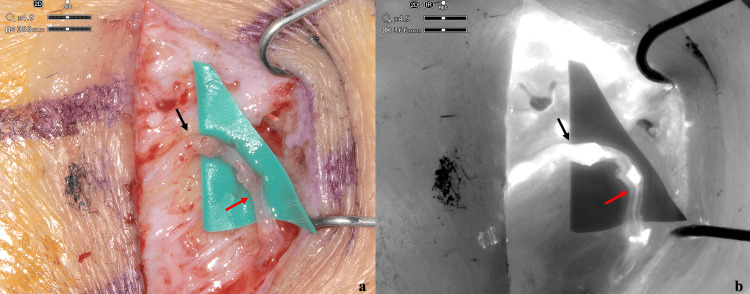
Intraoperative picture of supermicrosurgical lymphaticovenular anastomosis with ORBEYE system. (a) The picture shows the supermicrosurgical lymphaticovenular end-to-end anastomosis with 12-0 nylon between the ectasic lymphatic vessel (black arrow) and the neighboring no-reflux venule (red arrow). (b) In the infrared mode, the patency of the anastomosis was confirmed by the flowing of fluorescence from the lymphatic vessel (black arrow) to the venule (red arrow).

In the groin area, we found lymphatic vessels and venules of similar caliber, while we observed a greater mismatch at the knee and leg level due to compensatory dilation of the lymphatic vessels. We performed a standard anastomosis at the level of the lower leg and one at the knee level, while the one at the groin was performed using the efferent lymphatic vessel-to-venous anastomosis (ELVA) technique [14,15]. All anastomoses were performed using an end-to-end technique and 12-0 nylon sutures. The ORBEYE system was used throughout the entire surgery, for both dissection and anastomosis. At the end of the procedure, we confirmed the patency of the anastomoses using fluorescence (Figure 2).

**Figure 3:**
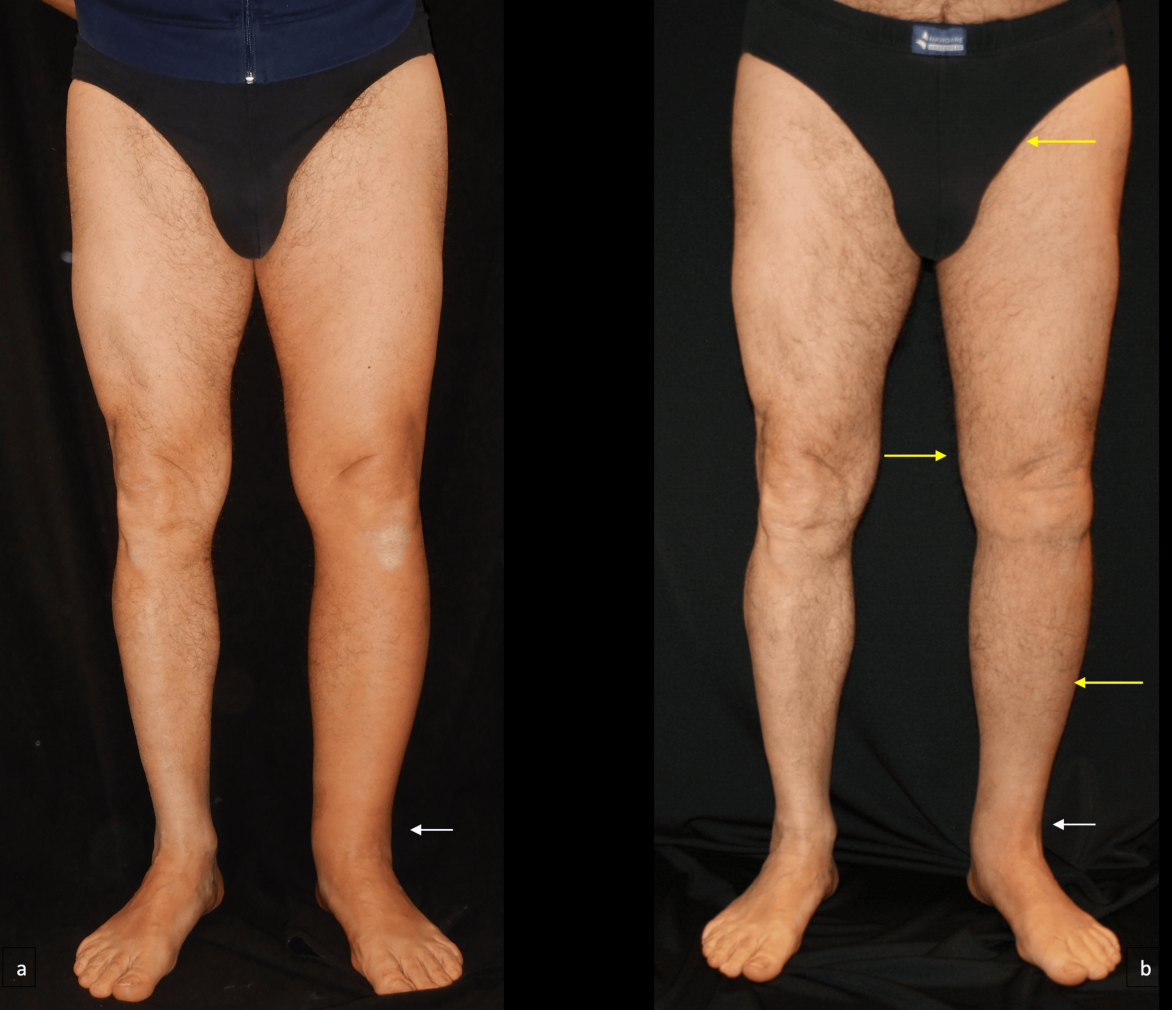
Lower limb lymphedema before and after surgery. These pictures show the anterior view of our patient, affected by left lower limb lymphedema, (a) before, and (b) one year after surgery. The yellow arrows at the level of the groin, knee, and leg indicate the three incision sites used for performing the lymphaticovenular anastomosis. The white arrows indicate the point of greatest improvement achieved after surgery at the level of the ankle.

The operative time was 247 minutes. No complications were encountered after the surgery. Conservative measures were optimized prior to the procedure with a course of complex decongestive physiotherapy and the subsequent use of a maintenance compression garment, following an already published protocol [16]. The same measures were continued after the procedure. The patient was treated with bandaging for two weeks and then re-evaluated together with the physiotherapist for the preparation of a new compression garment. The mean circumference of the limb decreased from 33.2 cm to 31.8 cm (Figure 3). The patient’s follow-up period was 18 months.

## Discussion

**Figure 4:**
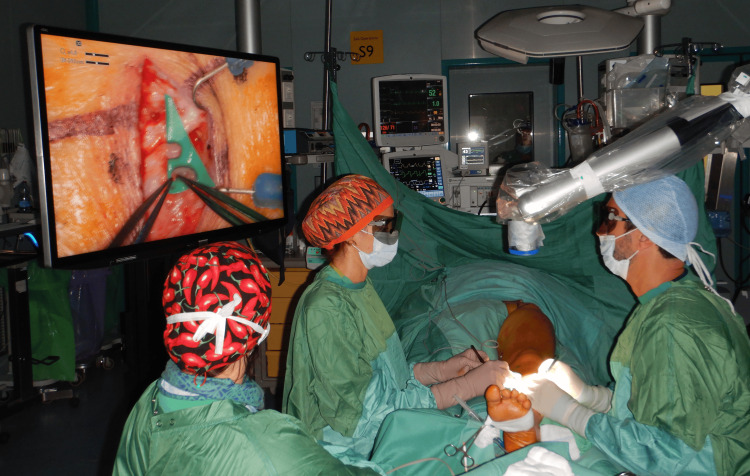
Intraoperative setup for lymphaticovenular anastomosis using the ORBEYE. The exoscope camera was placed above the surgical field which was displayed on the 55-inch 4K-3D monitor placed ahead of the operator. The monitor in combination with the 3D polarized glasses worn by the surgical staff showed a 3D image of the surgical field. ORBEYE has allowed a potentially superior visualization of lymphatic and vascular structures while maintaining an ergonomic posture.

LVA is the most commonly used surgical procedure in the field of lymphatic surgery. Many requirements are needed for a successful operation. Among these, the experience of the microsurgeon, the appropriateness of the instruments, and a high magnification system to perform the anastomosis with vessels smaller than a millimeter are of primary importance. The 4K-3D ORBEYE is an exoscope that visualizes the surgical field from above using a flexible and fully maneuverable 3D camera connected to a processor. The view of the surgical field is projected onto a 55-inch 4K-3D monitor positioned in front of the operator and a 31-inch 4K-3D monitor positioned behind the operator for assistance. The use of polarized 3D glasses in combination with the system achieves a three-dimensional image visualization (Figure 4) [9].

Previous studies have demonstrated the feasibility and effectiveness of applying the 3D exoscope to microanastomoses, with superior ergonomics compared to the traditional microscope [1,8-10]. A randomized controlled study compared the learning curve between ORBEYE and the traditional microscope in performing the dissection of a grape, demonstrating comparable performance [8]. In this report, we have described our experience in using ORBEYE for LVA in a case of lower limb lymphedema. ORBEYE offered a high-quality three-dimensional visualization of the surgical field, comparable to a traditional microscope in terms of resolution and illumination, with the added perception of depth. The ability to set the fluorescence mode for ICG allowed for easy visualization of lymphatic vessels. The easy maneuverability of the system allowed the surgeon to adjust the orientation of the ORBEYE camera on the surgical field depending on the site of the anastomosis, also leaving more free space around the operating table. The greater maneuverability compared to a traditional microscope was particularly useful in the anatomical site at the medial surface of the knee, where, in standard conditions, it is necessary to move the microscope in almost tangential planes and assume uncomfortable positions for an extended period. The increased freedom of movement of ORBEYE and the presence of an external monitor avoids the need to look through the eyepieces, as with the microscope, and allows for a more ergonomic position. Although we do not have objective data, we had the impression that the operative time was slightly prolonged. This fact could be attributed to the repeated movements of ORBEYE needed during dissection, the latency required for focusing, and the surgeon's adaptation to viewing on the monitor, which requires moving the hands while looking away from the operating field. This limitation is in line with previous studies that have used exoscopes for microsurgery, emphasizing the need for targeted training to adapt the surgical time to that of traditional microscopes [6]. ORBEYE represents a useful evolution of intraoperative magnification systems and can be considered adaptable to perform lymphovenous anastomoses after a learning curve. Its features and 3D vision can offer an advantage, especially in lymphatic supermicrosurgery. In fact, the 3D vision of a standard binocular microscope is limited by the high magnification required to perform procedures on lymphatic structures due to their small size. The exoscope enhances three-dimensionality through image processing, the external monitor, and 3D glasses. Further studies are needed to confirm the results of our experience and the applicability of the 4K-3D ORBEYE system to LVA. Considering the greater ergonomics and comparable magnification capability, compared to the standard microscope, we expect that it will become possible to apply exoscopes extensively to lymphatic surgery, particularly for anatomical sites that are uncomfortable to treat with a traditional microscope, such as the medial surface of the knee, or for obese patients [17-20].

## Conclusions

The ORBEYE represents a useful evolution of intraoperative magnification systems with a greater ergonomics and a non-inferiority magnification compared to traditional microscopes. Despite our findings need further studies to confirm the feasibility, the 4K-3D ORBEYE can be considered suitable to perform LVA after a fair learning curve.
